# Impact of malaria morbidity on gross domestic product in Uganda

**DOI:** 10.1186/1755-7682-5-12

**Published:** 2012-03-22

**Authors:** Juliet Nabyonga Orem, Joses Muthuri Kirigia, Robert Azairwe, Ibrahim Kasirye, Oladapo Walker

**Affiliations:** 1 World Health Organization, Country Office, Kampala, Uganda; 2 World Health Organization, Regional Office for Africa, Brazzaville, Congo; 3 Economic Policy Research centre, Makerere University, Kampala, Uganda; 4 Inter-country Support Team, World Health Organization. Regional Office for Africa, Harare, Zimbabwe

## Abstract

**Background:**

The burden of malaria is a key challenge to both human and economic development in malaria endemic countries. The impact of malaria can be categorized from three dimensions, namely: health, social and economic. The objective of this study was to estimate the impact of malaria morbidity on gross domestic product (GDP) of Uganda.

**Methods:**

The impact of malaria morbidity on GDP of Uganda was estimated using double-log econometric model. The 1997-2003 time series macro-data used in the analysis were for 28 quarters, i.e. 7 years times 4 quarters per year. It was obtained from national and international secondary sources.

**Results:**

The slope coefficient for Malaria Index (M) was -0.00767; which indicates that when malaria morbidity increases by one unit, while holding all other explanatory variables constant, per capita GDP decreases by US$0.00767 per year. In 2003 Uganda lost US$ 49,825,003 of GDP due to malaria morbidity. Dividing the total loss of US$49.8 million by a population of 25,827,000 yields a loss in GDP of US$1.93 per person in Uganda in 2003.

**Conclusion:**

Malaria morbidity results in a substantive loss in GDP of Uganda. The high burden of malaria leads to decreased long-term economic growth, and works against poverty eradication efforts and socioeconomic development of the country.

## Background

Malaria is endemic in 95% of Uganda, the remaining 5% of the country, mainly the highland areas, being epidemic prone. It is estimated that 93% of the total population in the country is at risk of malaria [1]. Although all four species of the malaria parasite exist in Uganda, *plasmodium falciparum*, which causes severe forms of malaria, is responsible for over 95% of cases. This parasite has shown increasing resistance to commonly used antimalarial medicines particularly Chloroquine and Sulfadoxine-Pyrimethamine (SP), as monotherapy and more recently in combination. In response to this, the country changed the malaria treatment policy to use of ACTs as first line treatment in 2005, whilst maintaining quinine as the second line treatment. Currently, data on ACT resistance in Uganda is not available.

Malaria contributes the major share of the disease burden with 39% of outpatient attendances and 35% of inpatient admissions being due to malaria [2]. In recent years there has been an increasing trend in clinically diagnosed malaria cases reported in the Health Management Information System (HMIS) (government and NGO health facilities) from 5 million cases in 1997 to 16.5 million cases in 2003. This translates into a 2003 incidence rate of 0.98 per person per year in children under 5 and 0.64 per person per year in older patients (based on HMIS data). The two major reasons for this increase are thought to be: the increased utilization that followed the abolition of user fees in the public sector, and increasing treatment failures due to drug resistance.

Various surveys indicate that approximately 60-80% of fever cases are treated in the informal and private sector. These figures translate into 65 million fever cases in 2003 treated as malaria. The prevalence rates for malaria parasitaemia (asymptomatic) range between 50% and 80% in young children, 20%-50% in older children and generally below 30% in adults. In Uganda, about 29-50% of outpatient visits at health facilities were attributable to malaria in 1999 [1]. This is a significant burden to the health system.

Malaria and malaria-related illnesses contribute a significant proportion (20-23%) of under five mortality. The estimated annual numbers of deaths from malaria range from 70,000 to 100,000. Malaria case fatality rate in 2001 was found to be 4.05% of inpatient cases [1].

The impact of malaria has been categorized from three dimensions, namely: health, social and economic. The health dimension is usually described in terms of life years lost to premature death, as well as the morbidity caused by the disease. The social dimension focuses on the coping strategies for the disease and also the hindrances to usual social participation. The economic dimension normally attempts to capture and present the impact of the two dimensions (health and social) into monetary terms. Broadly, the economic dimension of disease burden focuses on three main types of effects, namely: direct, indirect and intangible effects. These effects are felt at both macro (national and community) and micro (household and individual) levels.

Direct costs of malaria are the costs incurred by government, donors, communities, households and/or individuals in relation to providing or seeking treatment for malaria or preventive actions against malaria. The indirect costs of malaria refer to the productivity losses due to illness or premature death. Malaria-related absenteeism, debility and mortality have a negative impact on the quantity and quality of work/production. Time lost to care for the sick increase the indirect costs of malaria. Intangible costs mainly refer to the anxiety, pain and suffering resulting from illness.

Although Uganda's economy has been steadily growing over the past two decades, there has been stagnant growth (3-5%) over the past few years [3]. Burden of malaria is a challenge to both human and economic development. With concerted effort, malaria has been successfully controlled and eliminated completely in some parts of the world. While it might not be possible to eradicate malaria in some tropical countries such as Uganda, it is possible to control the disease and reduce its burden. Possibly, not so much effort has gone into interventions aimed at preventing and controlling malaria because the real impact (burden) of the disease has not been quantified and documented. In other words, the benefits of reduced malaria transmission are not well documented and hence appreciated.

The specific objective of this study was to estimate the impact of malaria morbidity burden on gross domestic product (GDP) in Uganda using the *production function *approach.

## Methods

**Figure 1:**
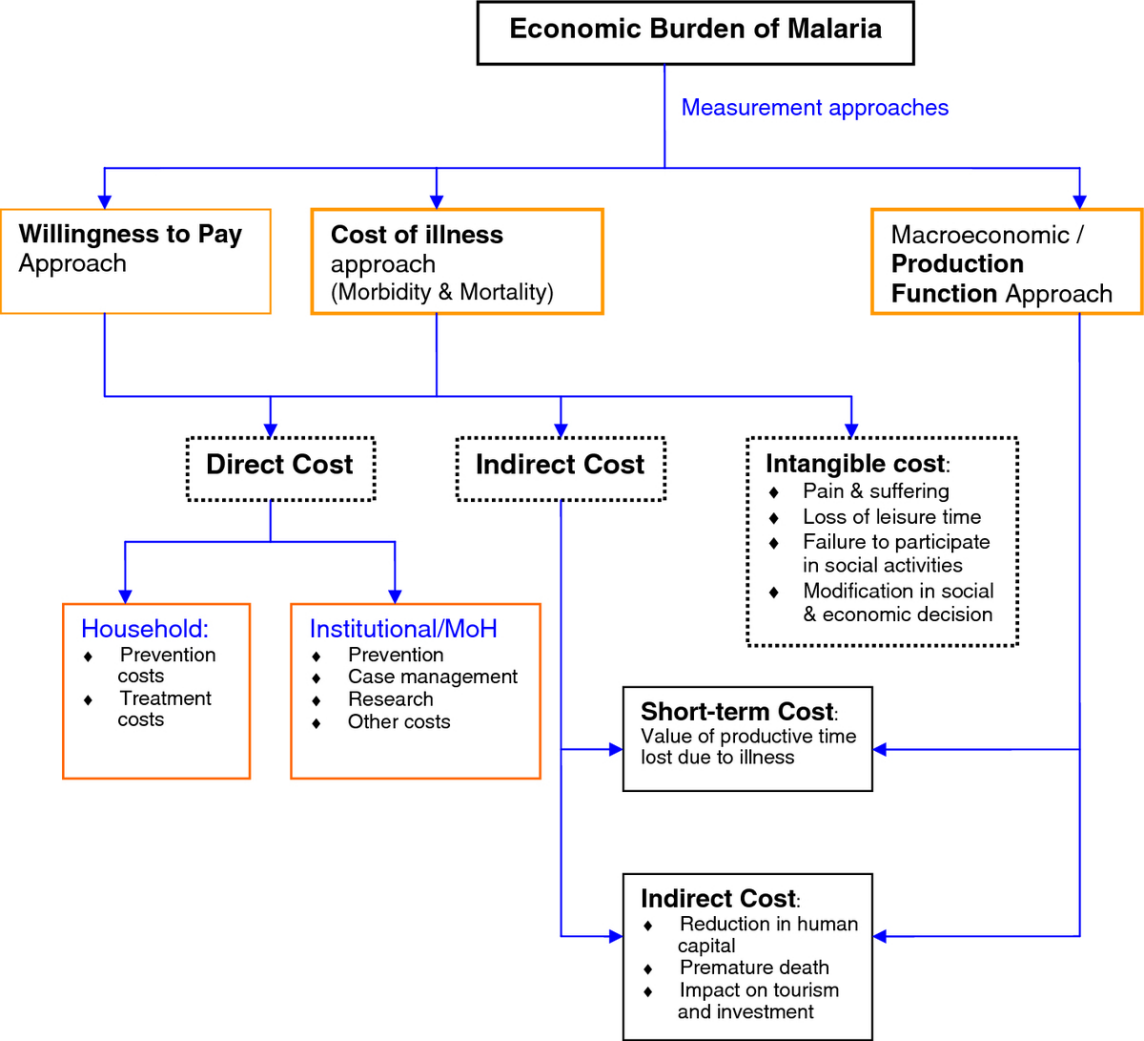
**Conceptual framework for economic burden of malaria**.

As depicted in Figure 1, there are mainly three methods of estimating the economic burden of any disease: (i) the production function approach that entails econometric modeling of the relationship between malaria disease burden and GDP; (ii) the total cost-of-illness approach; and (iii) the willingness-to-pay approach. The conceptual framework for the study, which illustrates the linkages of these approaches and is measured, is illustrated in Figure 1. This study employed the production function approach.

While the cost of illness approach provides insights into the economic burden of malaria at the household level, it does not capture large economic costs such as lost productivity, increased government expenditures and reduced private earnings. For instance, individuals may continue working while ill however with reduced capacity; others may out rightly abscond from duty, yet others may miss work in order to provide care for the sick. Previous studies examining the economic impact of health status in general and in particular malaria are cross-country analyses [4,5] and few of them are country specific. However, there is consensus that poor health negatively affects a country's overall productivity and aggregate output.

Following previous studies analyzing the effect of health on economic growth [5-9] the traditional production function approach is employed. The production function captures the aggregate relationship between inputs and outputs and thus intuitively provides the economy's state of technology.

The key components of GDP, which is the measure of aggregate output of the economy include: personal consumption; government expenditure, private investment, capital, and net exports (i.e. exports minus imports). As illustrated by Sachs and Malaney [10], the incidence of malaria can affect aggregate output through its effects on personal consumption, government expenditures and private investments. The effects of malaria on private consumption are indirect, via the increased labour turnover path and consequently reduced earnings. Government expenditures on malaria include: subsidies for the cost of treatment in public hospitals, the cost of prevention such as vector control, the cost of training health personnel and expenditure on malaria research. The impact on private investments is mainly through private medical costs such as expenditure on treatment and prevention of malaria, which can reduce private savings and hence private investments. However, despite the above links through which malaria incidence can impact on economic growth, the mechanisms through which the effects are transmitted are not empirically known in Uganda. Thus, this study attempts to empirically establish the ways through which malaria affects GDP in Uganda. The effect of malaria morbidity on GDP is captured by the economy's production function as follows:

(1) GDP=f(K,L,HK,I,A,T,M)

Where: GDP = real per capita gross domestic product; K = physical capital stock; L = labour (persons economically active); HK = human capital; I = inflation or general price changes; A = agriculture (the most important sector in Uganda); T = openness to trade of the economy; and M = number of reported malaria cases per 100,000 individuals. Equation (1) shows the effect of malaria on GDP holding other explanatory variable variables constant. We expect a negative relationship between malaria morbidity and GDP since the disease imposes an economic cost both to private individuals and government as earlier mentioned. Assuming the standard linearity assumption between explanatory variables in a regression is violated; equation (1) can be expressed in its Cobb Douglas production function form as:

(2) $$GDP=(AK^{\beta 1}L^{\beta 2}HK^{\beta 3}I^{\beta 4}T^{\beta 5}A^{\beta 6}M^{\beta 7}\varepsilon^{i})$$

Equation (2) can be transformed into its log linear (double-log) functional form as:

(3) $$\ln\;GDP=A+\beta_{1}\ln\;K+\beta_{2}\ln\;L+\beta_{3}\ln\;HK+\beta_{4}\ln\;I+\beta_{5}\ln\;gT+\beta_{6}\ln\;A+\beta_{7}\ln\;M+\varepsilon$$

A description of the model variables, data sources and methodology for estimating the effects of malaria and other explanatory variables on GDP is provided below.

### Dependent variable: per capita GDP

The dependent variable in the model is the per capita GDP, which is aggregate output divided by the total population. Since per capita GDP is only available at an annual basis, data splicing was undertaken to acquire a quarterly series. Splicing of GDP per capita was based on the real producer price of coffee and the real import price index following the methodology proposed by Henstridge [11]. Annual real per capita GDP data were obtained from 2003 Statistical Abstract and it is measured in US$ and constant 1997/1998 prices [12-15].

### Independent variables

#### Malaria index

The definition of malaria index has varied across studies, mainly due to data availability. While McCarthy *et al. *[9] consider malaria index as the population exposed to malaria morbidity, Gallup and Sachs [4] define it as the intensity of malaria within a given area and population. However, the latter approach is not employed due to the unavailability of geographical data showing the population at risk. Thus, the index based on McCarthy *et al. *[9] methodology is used. Formally, the malaria index is derived as expressed in equation (4).

(4) $$M=\left(\frac{Total\;malariacases\;reported}{Total\;population}\right)\times 100,000$$

The number of malaria cases is assumed to be the same for all persons regardless of age. Data on the number of malaria cases was only available from 1997 and this limits our ability to use a sufficiently long time series on annual basis. Instead, the models are estimated using quarterly data. However, also quarterly data on malaria cases was available for only seven years (1997-2003); consequently, our time series was of 28 quarters. Data for all malaria cases reported from every health facility in Uganda for each quarter were obtained from the health management information system (HMIS) database of the Ministry of Health [16]. The study recognises and acknowledges the limitations of using facility-based reporting which underestimates the total number of malaria cases in the country. The population data are based on the 2002 population and housing census and are obtained from the Ministry of Finance and Economic Development's Background to the Budget 2004/05 [13].

#### Capital

Capital is an input into the economy's production function. Increased capital accumulation leads to increased economic growth. However, as earlier mentioned increased malaria incidence affects capital accumulation indirectly; through the effects on private savings. Reduced savings negatively affects private investments and ultimately capital accumulation. Overall, we expect a positive effect of capital stock on aggregate output. A proxy - the share of gross fixed capital formation in GDP, captures the capital stock. The annual gross fixed capital series is obtained from the 2004/2005 Background to the budget from the Ministry of Finance Planning and Economic Development [13]. Due to estimation limitations, annual series is not spliced thus all quarters in a particular given year have the same values.

#### Labour

Malaria can affect labour through reduced work performance, increased labour turnover and through labour loss in cases where the disease results in mortality of the affected person. On the other hand, labour force positively impacts on aggregate output. The data for total labour force were obtained from the 2004 Africa Development Indicators of the World Bank [3]. Labour enters the model as the percentage of economically active persons in the total population.

#### Human capital

In addition to the physical capital variable, an indicator for human capital is included in the regression as suggested in the economic growth literature [17]. We expect a positive effect of human capital on GDP. Due to low secondary school enrolment gross primary enrolment is used as a proxy for human capital. The data for primary enrolment is obtained from the Uganda Bureau of Statistic's Statistical Abstract of 2003 [12]. Based on the assumption that enrolments do not change significantly within a year, all quarters in a particular year are assigned the same values.

#### Openness

Also as suggested in the growth literature for example in Millner and Upadhyay [18], a variable for trade orientation or openness is included in the regressions. In developing countries, which heavily depend on imports, the effect of trade on GDP can either be: negative if imports dominate trade, or positive if exports predominate trade. The share of total exports and imports in GDP is used to capture openness to trade. The quarterly imports and export data were obtained from various issues of the Bank of Uganda Quarterly Reports [15].

#### Inflation

Also included in the regression is a variable to capture economic stability. We expect a negative relationship between aggregate output and inflation. The variable is captured by consumer price index and the quarterly series is obtained from various issues of the Bank of Uganda Quarterly Reports (100 = 1997/98) [15].

#### Agriculture

Finally, because Uganda is an agricultural country, a variable accounting for this very important sector of the economy was included. The share of both monetary and non-monetary agriculture in GDP captures the agriculture variable. This data is obtained from the Ministry of Finance and Economic Development's Background to the Budget 2004/05 [13].

### Data

Data sources for each of the dependent and explanatory variables have been explained under each variable heading above. It is important for the reader to remember that this is a study based on secondary time series data for 28 quarters, i.e. 7 years times 4 quarters per year. The data was generally available at a highly aggregate level for the country as a whole, and thus, it does not tell much about the individual or micro units. For example, the Malaria Index is based on the total number of malaria cases recorded in all health facilities during each quarter as contained in the HMIS database. Since this is data reported from all health facilities in the country there is no sampling involved. Similarly, the data on macro-economic variables are for the entire country.

### Estimation strategy

Parameter estimates for equation (3) are obtained using the OLS method. A number of tests were undertaken to validate the regression results. Misspecification tests undertaken included: tests for seasonality - since the data was interpolated into a quarterly series, and tests for normality. In addition, tests for multicorrelinearity are carried out using the variance inflation factor (VIF) procedure. Finally, the estimated regression is tested and corrected for serial autocorrelation. The coefficient estimates are interpreted as elasticities, that is the percentage change in GDP for a given change in the explanatory variable. According to Gujerati [19] slope coefficients or marginal effects parameters are obtained using the following formula: $\left[\left(\bar{GDP}pc/\bar{IV_{i}}\right)\times \beta_{i}\right]$, where: $\bar{GDP}$ is the average of dependent variable, i.e. GDP; $\bar{IV_{i}}$ is the mean of i^th ^independent (explanatory) variable; *β_i _*is the elasticity of the logarithm of a specific independent variable. For example, let us assume that the per capita GDP for Uganda is US$242.06 per year; and average gross primary school enrolment is 5897271 children. The slope for gross primary school enrolment is obtained as follows: [(242.065897271)  ×  0.9118] =0.0000374.  The interpretation of the slope coefficient 0.0000374 is that if gross primary school enrolment increases by one child, GDP per capita on average increases by US$0.0000374 per year.

### Limitation of the current study

(a) *Weaknesses of facility-based data: *The data on malaria cases are from the HMIS database of the Ministry of Health. This data is gathered through facility-based reporting. It underestimates that total number of malaria cases since it does not capture people who had malaria but did not seek care at health facilities. Therefore, we may have underestimated the total economic burden of malaria morbidity.

(b) *Malaria-related morbidity among children under five years of age: *Approximately, 60% of malaria cases in Uganda are less than five years old. Those children were not included in our analysis. However, it could be argued that even though such children are not currently making an economic contribution, sickness may impact negatively on their physiological growth and intellectual development, and hence, future productivity that cannot be easily captured in static analysis.

(c) *Omission of economic burden of malaria-related mortality: *This study attempted to estimate the loss in GDP due to malaria morbidity and not the total economic cost of malaria. Therefore, the current study omits the economic burden of malaria mortality.

(d) *Use of data from different secondary sources: *The data on different variables (malaria index, capital, labour, gross primary enrolment, openness, inflation, agriculture) were obtained from different secondary sources. This is potentially a problem bearing in mind that errors of measurement are inevitable whenever we measure variables of any relationship and collect relevant data. Since we did not know what errors may have been lurking various datasets, it is difficult to tell how they might have impacted the estimates reported in this paper.

(e) *Use of "old" data: *The data for 1997-2003 may be argued to be a bit old. Due to dearth of research resources it was not possible to collect data for the period after 2003. The age of estimates may not matter much if one recalls that the purpose of cost-of-illness studies (like the one reported in this paper) is not to inform priority-setting but rather to raise awareness of the likely economic impact of a public health problem. Of course, such an argument does not obviate the need for further research in future to estimate the impact of malaria morbidity (and mortality) on GDP using more recent data.

## Results and discussion

Table 1 provides descriptive statistics for all the variables used in the study. The dependent and independent variable means reported in Table 1 were used in calculating the slope coefficients or marginal effects in Table 2. The regression estimates based on equation (3) are presented in Table 2. Model (1) presents the results of the estimation before correcting for serial autocorrelation. In this model the Malaria index is statistically significant in the hypothesized direction at the 95% level of confidence. However, after correcting for autocorrelation (Model 2), the significance for the malaria index reduces. It becomes significant only at the 90% level of significance. The following discussions are all based on Model 2. The overall fit of the model is very good with the adjusted coefficient of determination (R^2^) of 0.93, implying that 93% of the variation in log of per capita GDP is explained by the regression equation.

**Table 1:** Mean and Standard Deviations of the model Variables

*Variable *	*Mean *	*Standard deviation*
GDP: Per capita GDP-US dollars	242.06	61.13
M: Malaria Index	5522.61	3343.88
K: Share of gross fixed capital formation in GDP (%)	19.04	1.49
L: Share of economically active persons in GDP (%)	49.08	0.43
HK: Gross primary school enrolment	5897271	1337912
T: Share of imports and exports in GDP (%)	8.07	1.36
I: Consumer price index (inflation)	109.59	9.75
A: Share of agriculture in GDP (%)	40.65	1.08

**Table 2:** Double-log regression results (log of per capita GDP was dependent variable)

*Explanatory variables*			*Model 1*			*Model 2*
	
	*Coefficient*	*'t' statistic*	*Marginal Effects*	*Coefficient*	*'t' statistic*	*Marginal Effects*
Capital (K)	0.0008	0.04	0.010	0.0006	0.03	0.007628
Labour (L)	0.8373	3.21*	4.12952	0.8185	3.10*	4.036799
Human capital (HK)	0.9118	5.43*	0.0000374	0.8953	5.22*	0.0000367
Inflation (I)	-0.411	-0.6	-0.907808	-0.360	-0.5	-0.79516
Trade (T)	-0.104	-8.3*	-3.1195	-0.106	-8.5*	-3.17947
Agriculture (A)	-0.165	-2.8*	-0.98253	-0.156	-2.6*	-0.92894
Malaria index (M)	-0.178	-2.0*	-0.007802	-0.175	-1.9**	-0.00767
Constant	-38.85	-2.5*		-38.27	-2.4*	
Durbin-Watson statistic		1.75			1.88	
R-squared		0.941			0.939	
Adjusted R-squared		0.921			0.918	
VIF		1.67			-	
Observations		28			28	

Note: *means that the variable has a statistically significant impact on GDP per capita at the 95% level. **statistically significant at 90% level

In Table 2 both the regression coefficients (elasticities) and the marginal effects coefficients are presented. Regression coefficients (elasticities) mean that if an explanatory variable *X*_1 _changes by one percent while the other explanatory variables *X_i _*are held constant, then dependent variable will change by *β_i _*percent. The slope coefficient indicates the change in the dependent variable (per capita GDP) associated with a one-unit increase in the independent variable in question holding constant all the other independent variables in the equation. Thus, slope coefficient (or marginal effect) for Malaria Index (M) of -0.00767 indicates that when malaria morbidity increases by one unit, while holding all other explanatory variables constant, per capita GDP decreases by US$0.00767 per year, i.e. the burden of malaria on per capita GDP.

With total GDP of Uganda Shillings (USh) 12,756,500 million in 2003 [20] the above reduction in per capita GDP due to malaria translates into a total of USh 97,842,355,000 (that is 0.00767 × 12.757 trillion). This GDP loss is equivalent to US$ 49,825,003 in 2003. This is a very substantive loss to GDP for a country such as Uganda. Dividing the total loss of US$49.8 million by a population of 25,827,000 [21] yields a loss in GDP of US$1.93 per person in Uganda in 2003.

This reduction in GDP could take a variety of forms such as: reduced labour performance and school attendance reduced household ability to save and invest, and modification of household economic decisions in response to the risk of contracting malaria, increased government expenditures on control and treatment of the disease.

Other significant determinants of GDP include labour, human capital, trade, and agriculture. The negative and highly statistically significant coefficient of the share of trade in GDP indicates that openness to trade has a very strong effect on GDP. The negative coefficient of the trade variable indicates that imports dominate international trade in Uganda. Indeed, for period 1997-2003 annual expenditure on imports averaged US$1240.3 million, which were more than twice the average exports earnings during the same period US$497.44 million [13].

All the other significant variables such as labour had the correct sign except agriculture; the most important sector in the Ugandan economy. The model indicates that a 1% increase in the share of agriculture reduces GDP by US$0.929. The above result may be explained by the fact that despite its key importance, agriculture's share in overall GDP has been on the decline from about 55% in 1990 to the current 38.6% in 2003 [13,14]. Thus, the continuous decline in agriculture during the period under study coupled with a positive increase in GDP may explain the negative relationship.

In Uganda 60.2% of the malaria cases occurred in individuals below 5 years. In comparison, a study in Nigeria noted that about 80% of the malaria cases were in the age group 0-14 years [22]. In this situation where most of the affected individuals are under 5 years, it is a challenge to quantify the economic impact of malaria, as this age group is not yet in gainful employment. However, malaria affects the education of these children and thus reduces future skilled human capital that would have future economic impact. According to Sachs and Malaney [10], malaria reduces long-term economic growth. Why? Sachs and Malaney explain that ".. risk-averse houses raise fertility by even more than expected mortality, in order to ensure a sufficiently high likelihood of the desired number of surviving children. This theory predicts that a high burden of malaria will lead to a disproportionately high fertility rate and an overall high population growth rate in regions of intense malaria transmission". This has been found by the two authors (among others) to lead to decreased investment in education per child leading to a reduction in future educated human capital and thus reduced GDP, a phenomenon known as the quantity-quality trade off [10].

This study also showed that each child enrollment in primary school increases future GDP prospects by about US$0.000036. Previous studies have shown that malaria reduces primary school enrollment of poor households in malaria endemic regions [10]. By affecting the education of the children, malaria reduces the GDP prospects of a nation.

Furthermore, Onwujekwe et al [22] found that majority of the households in their Nigerian study were unemployed with about only 10% being employed or in self-employment. The fact that people are not in formal employment poses methodological challenges on the estimation of productivity losses. Thus it may be difficult to monetize the time lost as a result of malaria. The estimation of indirect costs (productivity loses) is therefore likely to result in an underestimated figure if the population studied is largely unemployed or has very low levels of income. It is critical that a true picture of the economic impact of this disease is measured since it has been found that in malaria endemic countries such as Uganda, malaria affects both the affluent and poor communities equally [4].

At the macroeconomic level, the indirect costs of malaria include those incurred as a result of productivity losses due to reduced labor performance. This is as a result of increased malaria morbidity and mortality, leading to increased absenteeism and labor turnover [10]. This translates into increased employment costs for recruitment, training and hence reduced productivity and GDP.

## Conclusion

This study has demonstrated that malaria has a statistically significant negative effect on per capita GDP of Uganda. Therefore, there is need for more investments into prevention and treatment of malaria. Such investments will contribute significantly to boosting the productivity of Uganda's population, and hence, increasing GDP.

Since the production function approach used in this study does not capture the full socioeconomic loss incurred by society due to malaria burden, there is need for further research to estimate the economic burden of malaria morbidity and mortality in Uganda, using total-cost-illness [23,24] and willingness-to-pay [25-27] approaches.

## Competing interests

The authors declare that they have no competing interests.

## Authors' contributions

JMO, JMK, RA, IK and OW contributed equally in design, analysis and writing of all sections of the manuscript. All authors read and approved the final manuscript.
